# Inflammatory exposures in early pregnancy and multidimensional sleep in late pregnancy: a prospective population-based study

**DOI:** 10.1016/j.bbih.2026.101354

**Published:** 2026-09-09

**Authors:** Fangxiang Mao, Milan Zarchev, Hong Sun, Yuchan Mou, Romy Gaillard, Isabel K. Schuurmans, Hanan El Marroun

**Affiliations:** a Department of Child and Adolescent Psychiatry, Erasmus University Medical Center, Rotterdam, the Netherlands; b The Generation R Study Group, Erasmus University Medical Center, Rotterdam, the Netherlands; c Department of Psychiatry, Erasmus University Medical Centre, Rotterdam, the Netherlands; d Department of Epidemiology, Erasmus University Medical Center, Rotterdam, the Netherlands; e Department of Pediatrics, Erasmus University Medical Center, Rotterdam, the Netherlands; f Department of Psychology, Education and Child Studies, Erasmus School of Social and Behavioral Sciences, Erasmus University, Rotterdam, the Netherlands

**Keywords:** Inflammatory response, Sleep, Pregnancy, Infection, Dietary inflammatory index, Cytokines

## Abstract

**Background:**

Sleep problems are common during pregnancy, and inflammation has been proposed as a potential underlying pathway. However, findings remain fragmented because most studies have focused on single inflammation-related exposures or individual sleep components. We therefore prospectively examined multiple inflammatory measures in relation to multidimensional sleep outcomes.

**Methods:**

Within the population-based Generation R *Next* cohort*,* inflammation was assessed in early pregnancy by (a) a composite score of self-reported infection-related conditions (n = 1134), (b) peripheral inflammatory markers including a cytokine index and hs-CRP (n = 432), and (c) dietary inflammatory potential indexed by the Dietary Inflammatory Index (DII) (n = 852). Sleep in late pregnancy was assessed by validated questionnaires including duration, latency, midpoint, quality, awakenings, difficulties initiating sleep, and daytime sleepiness. Associations were examined using Bayesian multivariate models that accounted for correlations among sleep components and estimated an overall sleep effect. We further tested the effect modification by body mass index.

**Results:**

Associations between inflammation in early pregnancy and sleep in late pregnancy differed across inflammation-related exposures. Self-reported infections were associated with poorer overall sleep (β = 0.06, 95%CrI = [0.03,0.09]). No associations were found for the cytokine index, hs-CRP, or DII and overall sleep. At individual sleep component level, associations were most evident for sleep quality and daytime sleepiness. Several associations between inflammation-related exposures and individual sleep components differed in direction and strength between women with overweight or obesity.

**Conclusions:**

Associations between inflammation and sleep during pregnancy were heterogeneous across inflammation-related exposures, sleep components, and BMI status. These findings support a multidomain inflammatory framework for understanding multidimensional sleep health during pregnancy.

## Highlights

• Inflammation-sleep associations varied across inflammatory domains.
• Early-pregnancy infections were associated with poorer late-pregnancy sleep.
• No associations were found for inflammatory diet or peripheral inflammatory markers.
• Associations were clearest for sleep quality and daytime sleepiness.
• Associations were generally stronger among women with overweight or obesity.

## 1 Introduction

Sleep problems are common during pregnancy, affecting approximately half of pregnant women (Yang et al., 2020), and represent clinically important behavioral symptoms. Short sleep duration and poor sleep quality have been linked to increased risks of gestational hypertension and diabetes in mothers, as well as adverse socioemotional development in offspring (Christian et al., 2019; Adler et al., 2021; Nevarez-Brewster et al., 2022; Zou et al., 2022). Importantly, sleep is not a unitary construct; it comprises distinct but interrelated components, including duration, timing, latency, daytime sleepiness, and quality. A multidimensional perspective may therefore be important for understanding both overall sleep vulnerability and component-specific patterns during pregnancy (Buysse, 2014).

The factors contributing to sleep problems during pregnancy remain incompletely understood. Sleep and inflammation are thought to be bidirectionally related (Engert and Besedovsky, 2025). Epidemiological research has more often examined sleep disturbance as a predictor of subsequent systemic inflammation or susceptibility to infection (Irwin et al., 2016; Prather and Leung, 2016), whereas experimental and mechanistic studies, particularly in animals, indicate that cytokines and other immune mediators can influence sleep homeostasis, arousal, circadian regulation, and daytime sleepiness (Besedovsky et al., 2019; Feuth, 2024; Ibarra-Coronado et al., 2015; Irwin, 2019). Prospective human evidence examining associations between earlier inflammation-related exposures and subsequently assessed sleep remains limited, particularly during pregnancy, when both immune activity and sleep change across gestation (Gigase et al., 2025; Haufe and Leeners, 2023; Yang et al., 2020).

Inflammation-related processes can be examined through conceptually distinct exposures, including self-reported infection-related conditions, peripheral inflammatory markers, and dietary inflammatory potential (Antonelli and Kushner, 2017; Minihane et al., 2015). These exposures reflect potential inflammation-activating health events, measured peripheral inflammatory activity, and an upstream dietary exposure that may shape chronic low-grade inflammatory regulation, respectively.Together, the three exposure classes capture different positions along an inflammation-related pathway, retain distinct interpretations, and provide complementary perspectives. Associations with sleep have been reported for each exposure class in general adult populations, older adults, and populations with obesity, although the direction of assessment and study designs have varied (Bjorvatn et al., 2023; Ferreira et al., 2025; Feuth, 2024; Fu et al., 2025; Hepsomali and Groeger, 2022; Irwin et al., 2016; Jiang et al., 2025; Kase et al., 2021; Prather and Leung, 2016; Toğuç et al., 2024). During pregnancy, the available studies are few and largely cross-sectional, and findings have been inconsistent even for the same exposure class and sleep component (Amare et al., 2022; Jimah et al., 2022; Okun et al., 2007; Tamanna et al., 2026; Wirth et al., 2022; Zhang et al., 2025; Zhu et al., 2020).

Existing pregnancy studies leave three key gaps. First, previous studies have generally examined one class of inflammation-related exposure at a time, leaving limited findings on the respective associations of conceptually distinct inflammation-related exposures with sleep when assessed within the same population and analytical framework. Moreover, studies examining each type of inflammation-related exposure have often relied on narrow indicators, such as COVID-19 infection (Amare et al., 2022), single peripheral inflammatory markers, such as C-reactive protein (CRP) or interleukin-6 (IL-6) (Okun et al., 2007; Zhu et al., 2020), or single anti- or pro-inflammatory food items, such as monounsaturated fat and vitamins (von Ash et al., 2023). These measures may not adequately capture broader infection-related conditions, inflammatory activity, or dietary inflammatory potential. Second, most studies are cross-sectional, limiting insight into the temporal ordering of inflammatory exposures and sleep outcomes. Third, potential modification by maternal body mass index (BMI) has rarely been examined, despite evidence that overweight and obesity may modify inflammation-related sleep vulnerability (Mathis and Shoelson, 2011; Rigobon et al., 2018; Tamanna et al., 2026).

Using a population-based prospective cohort, we therefore examined whether three conceptually distinct inflammation-related exposures assessed in early pregnancy—self-reported infection-related conditions, peripheral inflammatory markers, and dietary inflammatory potential—were associated with sleep components assessed subsequently in late pregnancy. We further tested whether these associations differed by maternal BMI. In a subsample with repeated assessments, we explored whether inflammatory exposures at preconception and in late pregnancy showed similar associations with sleep in late pregnancy. We hypothesized that higher inflammation-related exposure would be associated with poorer sleep, and that the magnitude and pattern of associations would vary across sleep components and BMI.

## 2 Methods and materials

### 2.1 Participants

This study is embedded within the Generation R *Next* Study, a population-based prospective cohort in Rotterdam, the Netherlands. The aim of this study was to identify preconception and early-pregnancy determinants of fertility, embryonic development, and childhood outcomes (Boxem et al., 2024).

Women were eligible to participate in the Generation R *Next* Study if they (1) were aged ≥18 years, (2) lived in Rotterdam (zip codes 3011 to 3099 and 3191 to 3196), and (3) planned to get pregnant within a year (preconception period) or were pregnant at any gestational age. The inclusion period was between August 9, 2017, and July 1, 2021. As participants could enroll multiple times (once per pregnancy), data were collected for each enrollment. A total of 4036 pregnancies were enrolled during the study period. Because participants could enroll preconception or any time during pregnancy, not all participants contributed data to all assessments.

**Fig. 1:**
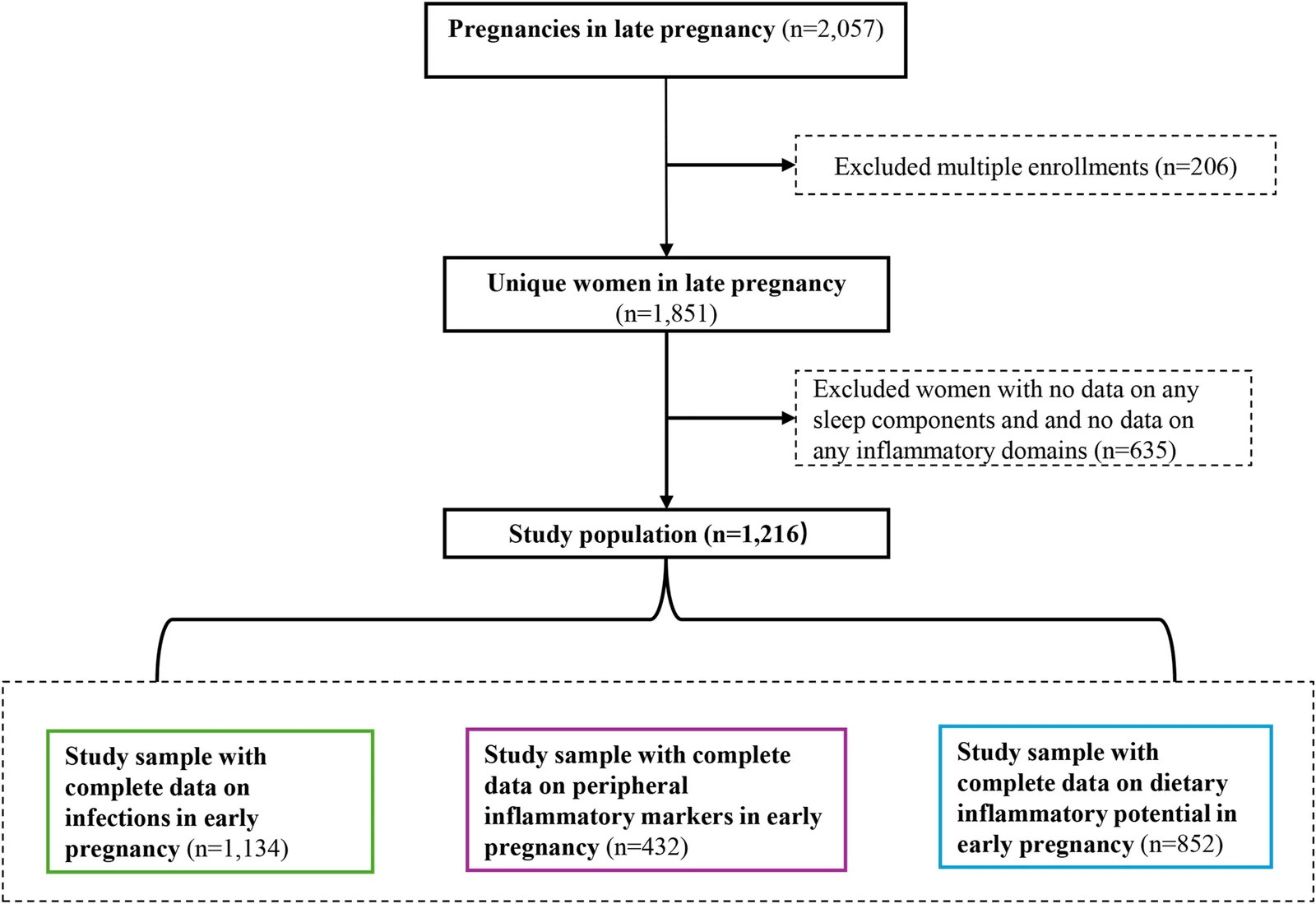
Flowchart of study sample inclusion and exclusion *Figure note:* After excluding women with multiple enrollments and those without any data on sleep components or inflammation-related exposures, 1216 unique women with a single pregnancy were retained in the study sample. Domain-specific study samples for self-reported infections (green, left), peripheral inflammatory markers (purple, center), and dietary inflammatory potential (blue, right) are shown separately. Sample sizes for peripheral inflammatory markers and dietary inflammatory potential were smaller because these data were available only for subsets of women with biological samples and food frequency questionnaires. (For interpretation of the references to colour in this figure legend, the reader is referred to the Web version of this article.)

The availability of each inflammation-related exposure and the corresponding analytical sample sizes are presented in Fig. 1. Peripheral inflammatory markers measured in biological samples were available for 1049 pregnancy enrollments. A randomly selected subgroup was invited to complete a food frequency questionnaire for assessing dietary inflammatory potential, resulting in dietary data for 1054 pregnancy enrollments (Schipper et al., 2025). Finally, the three classes of inflammation-related exposures were examined in domain-specific subsamples.

The present analyses used inflammation-related exposures assessed earlier in pregnancy and sleep assessed at the late-pregnancy follow-up. A total of 2057 pregnancies were invited to participate in the late-pregnancy assessment (Fig. 1). To ensure independence of observations, we retained one pregnancy per participant, yielding 1851 unique women in late pregnancy. When multiple pregnancies were available for the same woman, we selected the pregnancy with the most complete exposure or outcome data; if completeness was identical, one pregnancy was selected at random. We then excluded participants with missing data on all sleep components and all inflammation-related exposures (n = 635), resulting in a final analytic sample of 1216 unique women. The domain-specific analytic samples included 1134 women for self-reported infection-related conditions, 432 for peripheral inflammatory markers, and 852 for dietary inflammatory potential.

### 2.2 Measures

#### 2.2.1 Self-reported infection-related conditions

Self-reported infection-related conditions were assessed using four questionnaire items in early pregnancy (10.0 ± 5.2 weeks). Women reported whether they had experienced gastrointestinal infections (diarrhea or enteritis), cystitis/pyelitis, or dermatitis during the current pregnancy, and whether they had experienced fever (>38 °C/100.4 °F) during the previous year. For each item, women reported whether the condition was present or absent. A summed infection score (range = 0–4) was calculated to reflect the total number of infection-related conditions (hereafter referred to as the infection score). Although this measure does not capture all possible infections and was not validated against clinical diagnoses, a shorter 2-item questionnaire-based infection score was moderately correlated with a broader 8-item questionnaire-based measure in a previous study (Pearson r = 0.60) (Suleri et al., 2024). This finding provides some support for its use as a summary of self-reported infection-related conditions, but not as a clinical measure of infection or systemic inflammation. In exploratory analyses, the full four-item infection measure was not available at other time points. Only fever was assessed at preconception and only infection-related symptoms in late pregnancy; time-point-specific infection scores were therefore constructed.

#### 2.2.2 Peripheral inflammatory markers

Peripheral inflammatory markers were measured from early-pregnancy blood samples (9.7 ± 2.2 weeks). Blood samples were centrifuged for 10 min, and serum was aliquoted into 250-μL polypropylene Micronics tubes and immediately stored at −80 °C. Markers included high-sensitivity C-reactive protein (hs-CRP), interleukin-1β (IL-1β), interleukin-6 (IL-6), interleukin-17A (IL-17A), interleukin-23 (IL-23), and interferon-γ (IFN-γ). hs-CRP was measured by the Department of Clinical Chemistry at Erasmus MC. IL-1β, IL-6, IL-17A, IL-23, and IFN-γ were assessed using the High Sensitivity T-cell Discovery Array 5-Plex (Millipore, St. Charles, MO, USA) at Eve Technologies on the Bio-Plex™ 200 system (Bio-Rad Laboratories, Inc., Hercules, CA, USA). Details on sample collection, laboratory procedures, data processing, and quality assurance have been described previously (Gigase et al., 2025).

To derive a composite cytokine measure, cytokine concentrations were log-transformed, z-standardized, and entered into principal component analysis separately at each time point, in line with our previous study (Gigase et al., 2025). The first principal component was extracted and used as the cytokine index, with higher scores indicating higher overall cytokine levels. The cytokine index was based on IL-1β, IL-6, IL-17A, IL-23, and IFN-γ, whereas hs-CRP was analyzed separately because it may show different patterns of change across pregnancy (Gigase et al., 2025). In exploratory analyses, hs-CRP and cytokines were also assessed at preconception, and cytokines were additionally assessed in late pregnancy. Corresponding cytokine scores were calculated for the preconception and late-pregnancy time points.

#### 2.2.3 Dietary inflammatory potential

Dietary inflammatory potential was assessed using the Dietary Inflammatory Index (DII) in early pregnancy (15.0 ± 6.1 weeks). Higher positive scores indicate a more pro-inflammatory diet whereas lower scores indicate a more anti-inflammatory diet (Shivappa et al., 2014a, 2014b). In the current study, DII was calculated from 26 dietary components derived from the Dutch semi-quantitative HELIUS food frequency questionnaire (FFQ), which assessed maternal dietary intake in the preceding four weeks (Beukers et al., 2015). Its construct validity has been examined using peripheral inflammatory biomarkers (Tabung et al., 2015). We used the R package *DietaryIndex* to calculate DII (Zhan et al., 2024). Details of FFQ processing, food parameters, and DII calculation are provided in the Supplementary Methods and Table S1.

#### 2.2.4 Sleep

Seven sleep components were assessed at late-pregnancy follow-up (27.3 ± 2.3 weeks), including sleep duration, sleep latency, sleep midpoint, sleep quality, difficulty initiating sleep, midsleep awakenings, and excessive daytime sleepiness. Sleep duration, sleep latency, and sleep midpoint were derived from the Munich Chronotype Questionnaire (MCTQ) (Roenneberg et al., 2003). The MCTQ includes six items assessing bedtime, time intending to fall asleep, sleep latency, wake-up time, time until getting out of bed, and alarm use, separately for workdays and work-free days. These sleep components were calculated using the following formulas:

Sleepduration=wakeuptime−timeintendingtofallasleep−sleeplatency

Sleeplatency=thevaluefromtheoriginalitem,“Ineed...minutestofallasleep”

$$Sleep\;midpoint=time\;intending\;to\;fall\;asleep+sleep\;latency+\frac{sleep\;duration}{2}$$

Weighted weekly averages were calculated as follows: (sleep on workdays×5 + sleep on work-free days×2)/7. Sleep measures on workdays and work-free days were moderately to highly correlated (Pearson r = 0.43–0.61 for duration, 0.63–0.64 for midpoint, and 0.73–0.81 for latency).

Sleep quality, difficulty initiating sleep, midsleep awakenings, and excessive daytime sleepiness were derived from the General Sleep Disturbance Scale (GSDS) (Lee and DeJoseph, 1992). The GSDS consists of 21 items covering seven subscales: sleep quality, quantity, difficulty initiating sleep, midsleep awakenings, early awakenings, use of sleep medications, and excessive daytime sleepiness. Participants reported the number of days in the past week on which they experienced these symptoms. Subscale scores ranged from 0 to 21 for sleep quality, 0–7 for difficulty initiating sleep and midsleep awakenings, and 0–49 for daytime sleepiness, with higher scores indicating poorer sleep in each component. The GSDS is valid and reliable in pregnant women (Coo et al., 2014).

#### 2.2.5 Confounders

**Fig. 2:**
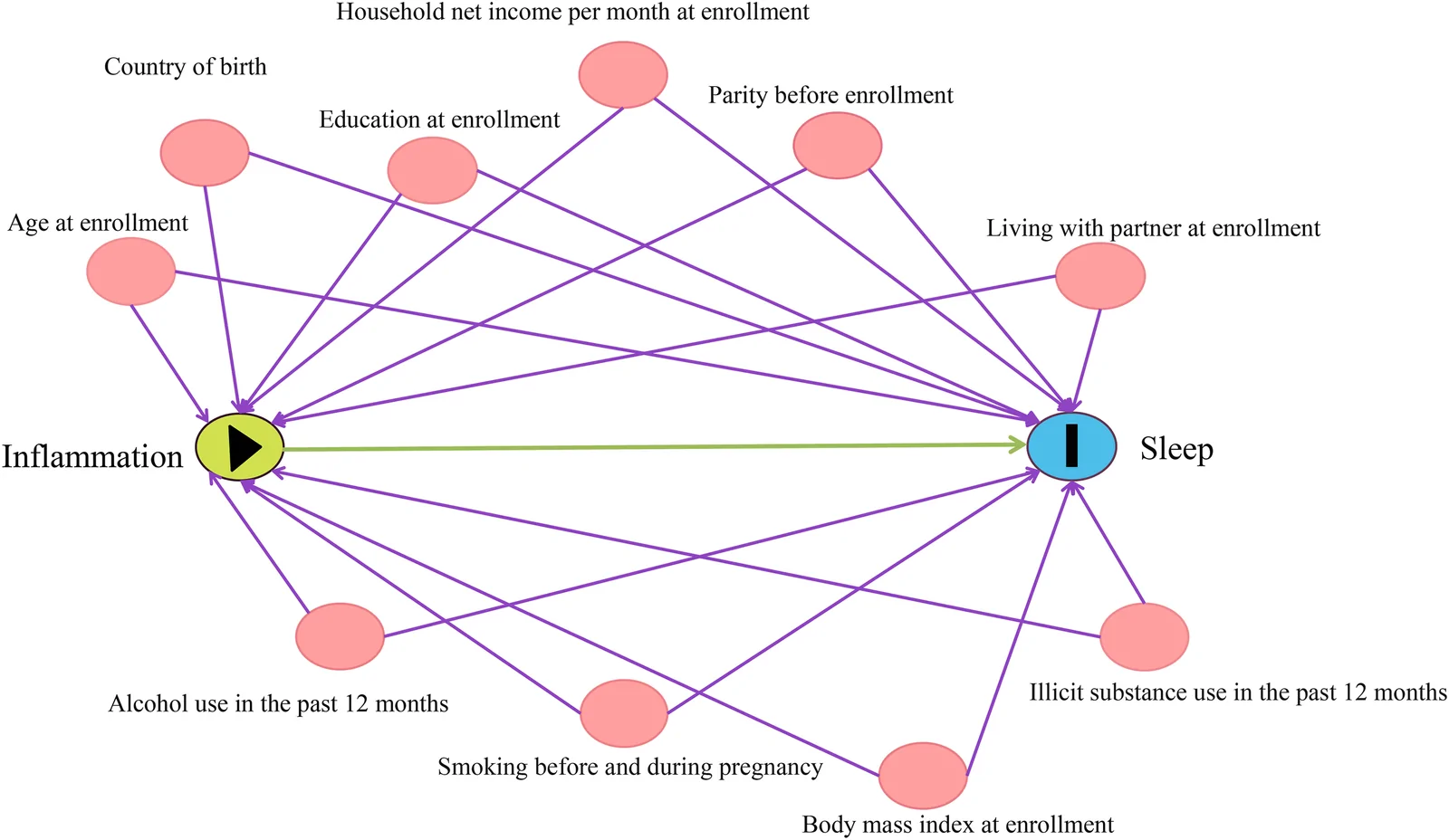
Directed acyclic graph *Figure note*: In the directed acyclic graph, the exposure (inflammation) is shown in yellow, the outcome (sleep) in blue, and common causes of the exposure and outcome in pink. The causal path from exposure to outcome is shown in green, and biasing backdoor paths are shown in purple. The DAG is simplified for clarity and therefore does not depict all interrelations among covariates (e.g., smoking and alcohol use are related to household income). (For interpretation of the references to colour in this figure legend, the reader is referred to the Web version of this article.)

Informed by published recommendations for covariate selection in inflammation and sleep research (O'Connor et al., 2009; Philippens et al., 2022), confounders were visualized in a directed acyclic graph (DAG, Fig. 2). At enrollment, we assessed sociodemographic and lifestyle factors. Sociodemographic factors included maternal age, maternal country of birth (the Netherlands vs outside the Netherlands), educational status (prevocational or vocational education, bachelor's or associate degree, or master's degree), household net income per month (<€3000 vs ≥€3000), living with a partner (yes [married and cohabiting, cohabiting but not married, or registered partnership with cohabitation] vs no [no partner and/or no cohabitation, widowed, married but living separately, divorced or separated, or other]), and parity before enrollment (0 = nulliparous; ≥1 = multiparous). Lifestyle factors included BMI at preconception (kg/m^2^), measured at the research center if women were enrolled before conception or self-reported if women were enrolled during pregnancy; self-reported smoking before and during pregnancy (never; yes, quit >3 months before pregnancy; yes, quit <3 months before pregnancy; yes, quit in the first trimester; or yes, smoked throughout pregnancy); self-reported alcohol use in the past 12 months (none vs any); and self-reported illicit substance use in the past 12 months (none vs any).

### 2.3 Statistical analyses

All analyses were conducted in R version 4.5.2. For the non-response analysis, demographic characteristics of responders and nonresponders were compared using independent t-tests, chi-squared tests, or Fisher's exact tests. Missing data in sleep outcomes and covariates were imputed using multiple imputation with 20 imputed datasets, and estimates were pooled using Rubin's rules (Rubin, 1987).

The main analyses were conducted separately for each inflammation-related exposure: self-reported infections, peripheral inflammatory markers (cytokine score and hs-CRP), and dietary inflammatory potential (DII). For each inflammatory exposure, we fitted a Bayesian multivariate model in *brms* with seven correlated sleep components jointly specified as outcomes. This approach accounted for intercorrelations among the sleep components within a unified analytic framework, thereby reducing multiple testing and providing a more integrated characterization of sleep. We selected this Bayesian multivariate framework, rather than a correlated-outcome structural equation model, because it offers greater flexibility in handling non-normal, differently scaled, and potentially non-linear sleep outcomes (Bürkner, 2017). In each model, residual correlations among sleep components were estimated to capture shared variance. Weakly informative priors were specified for all parameters, including normal(0, 20) priors for regression coefficients, Student-t priors for intercepts and scale parameters, and Lewandowski–Kurowicka–Joe (LKJ)(2) prior for the residual correlation matrix. Non-linearity was assessed using spline terms, which did not improve model fit. In accordance with Bayesian inference, we reported posterior estimates and 95% credible intervals (CrIs) from the Bayesian multivariate models for each sleep outcome. To summarize associations across sleep components, we calculated an overall sleep effect by averaging standardized coefficients across sleep components. All confounders identified through the DAG were included in the main analyses.

In secondary analyses, we examined effect modification by pre-pregnancy BMI by including an interaction term between each inflammation-related exposure and continuous BMI (Wirth et al., 2022). For visualization of notable interactions, predicted associations were plotted for women with and without overweight or obesity, using a BMI cutoff of 25 kg/m^2^.

In sensitivity analyses, we tested the robustness of results by (1) dichotomizing the infection score into “no infection” (score = 0) and “any infection” (score>0); (2) additionally adjusting total energy intake in DII analyses; (3) refitting selected sleep outcomes using alternative distributions, including ordinal model for midsleep awakenings and negative binomial model for difficulty initiating sleep; and (4) conducting leave-one-component-out analyses for all three inflammation-related exposures to assess whether observed associations were driven by specific infection conditions, peripheral inflammatory markers, or dietary components rather than the composite indices.

In exploratory analyses, we reran the main models for inflammatory exposures assessed at preconception and in late pregnancy in relation to sleep in late pregnancy to explore timing-specific associations.

## 3 Results

**Fig. 3:**
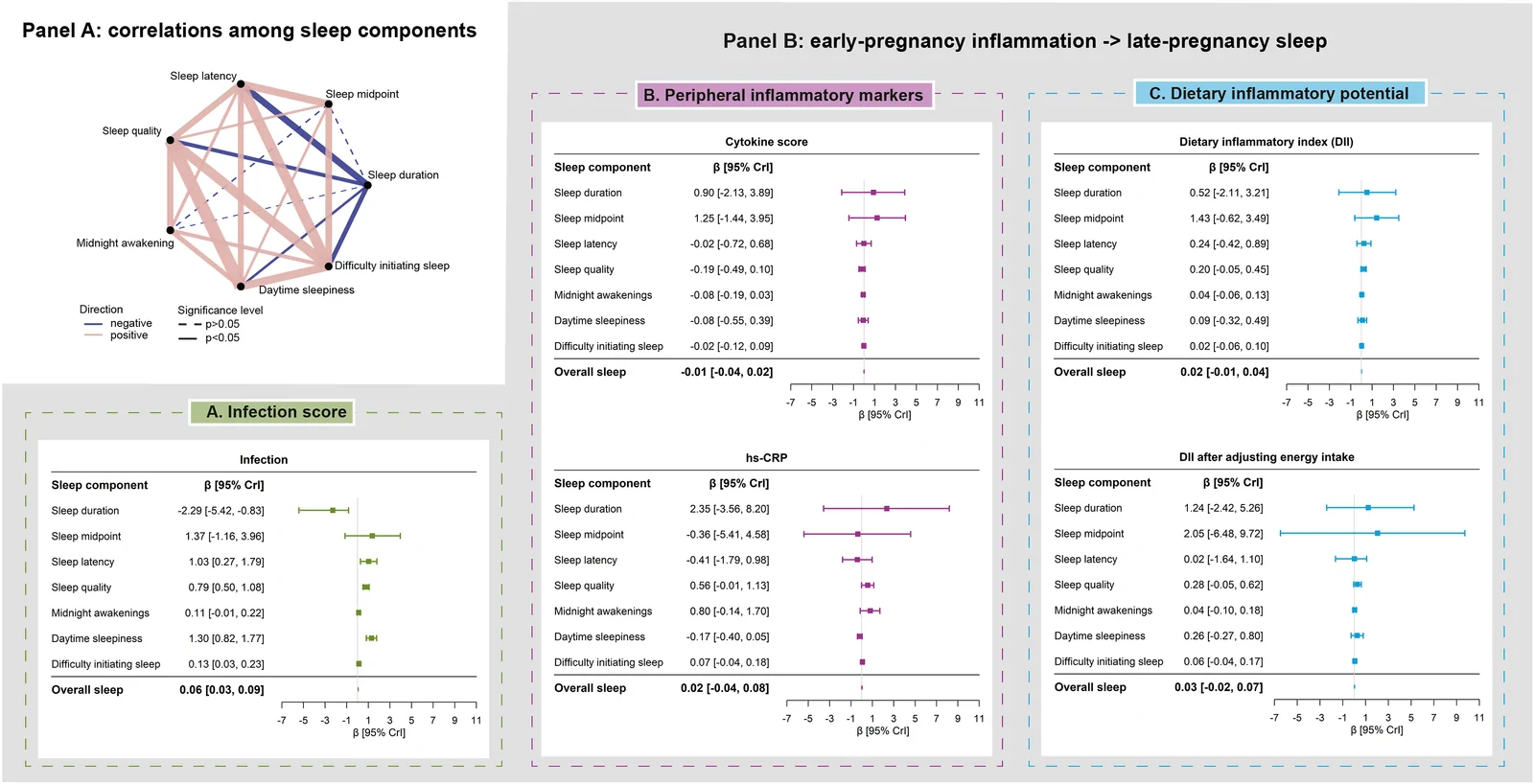
The temporal association between inflammation in early pregnancy and sleep in late pregnancy *Figure note:* Panel A shows raw correlations among the seven sleep components, without adjustment for covariates or inflammatory exposures. In Panel A, blue lines indicate negative correlations and pink lines indicate positive correlations; solid lines denote p < 0.05 and dashed lines denote p ≥ 0.05; line width is proportional to the magnitude of the correlation coefficient. Panel B shows posterior estimates (β) and 95% credible intervals (CrIs) for prospective associations between three domains of inflammatory exposures in early pregnancy and sleep in late pregnancy: infection score (A, green, n = 1134), peripheral inflammatory markers (B, purple, n = 432), and dietary inflammatory potential (C, blue, n = 852). Estimates were obtained from Bayesian multivariate models with seven correlated sleep components jointly specified as outcomes, with each inflammatory exposure entered separately. The overall effect was summarized by averaging standardized posterior regression coefficients across sleep components within each posterior draw. Models were adjusted for maternal age at enrollment, maternal country of birth, maternal education, household net income per month, living with a partner, parity before enrollment, body mass index, smoking before and during pregnancy, alcohol use, and illicit substance use. (For interpretation of the references to colour in this figure legend, the reader is referred to the Web version of this article.)

Women included in the study were aged 32.0 ± 4.1 years at enrollment (n = 1216). Nearly half had a master's degree and 66.2% were born in the Netherlands (Table 1). Correlations among sleep components ranged from weak to moderate, supporting the use of a multivariate framework while indicating that the components captured partly distinct aspects of sleep (Fig. 3, Panel A), whereas correlations among the three inflammation-related exposures ranged from −0.04 to 0.22. Compared with nonresponders, responders were more likely to have a higher education level, higher household net income per month, and to be nulliparous before enrollment (Table S2).

**Table 1:** Sample characteristics of women included in the analytic sample.

	Sample characteristics (n = 1216)
**Sociodemographic characteristics**	
Age at study enrollment, mean ± SD	32.0 ± 4.1
Country of birth	
The Netherlands	796 (66.2%)
Outside the Netherlands	406 (33.8%)
Education, No. (%)	
Prevocational or vocational	266 (22.3%)
Bachelor's or associate degree	355 (29.7%)
Master's degree	574 (48.0%)
Household net income per month, No. (%)	
<€3000	197 (18.6%)
≥ €3000	863 (81.4%)
Living with partner, No. (%)	
Yes	1047 (88.5%)
No	136 (11.5%)
Parity before enrollment, No. (%)	
0, nulliparous	838 (70.1%)
≥ 1, multiparous	357 (29.9%)

**Psychosocial and lifestyle characteristics**	
Body mass index at preconception (kg/m^2^), mean ± SD	24.0 ± 4.3
Smoking before or during pregnancy, No. (%)	
Never	615 (56.7%)
Yes, quit >3 months before pregnancy	243 (22.4%)
Yes, quit <3 months before pregnancy	81 (7.5%)
Yes, quit smoking in the first trimester	110 (10.1%)
Yes, smoked the whole pregnancy	35 (3.2%)
Alcohol use in the past 12 months, No. (%)	
None	235 (20.1%)
Any	933 (79.9%)
Illicit substance use in the past 12 months, No. (%)	
None	1100 (90.5%)
Any	116 (9.5%)

*Table Note:* Baseline characteristics of the study sample were summarized as n (%) or mean ± standard deviation (SD), as appropriate. Percentages of missing data ranged from 0 to 12.8% across variables. Household net income per month had the highest proportion of missing data.

### 3.1 Self-reported infections and sleep

Higher infection scores in early pregnancy were associated with poorer overall sleep in late pregnancy (β = 0.06, 95%CrI = [0.03,0.09]). In addition, higher infection scores were associated with longer sleep latency (β = 1.03, 95%CrI = [0.27,1.79]), poorer sleep quality (β = 0.79, 95%CrI = [0.50,1.08]), greater daytime sleepiness (β = 1.30, 95%CrI = [0.82,1.77]), and greater difficulty initiating sleep (β = 0.13, 95%CrI = [0.03,0.23]) (Fig. 3A). No clear associations were observed for sleep duration, midpoint, and midsleep awakenings (all 95%CrIs included the null).

**Fig. 4:**
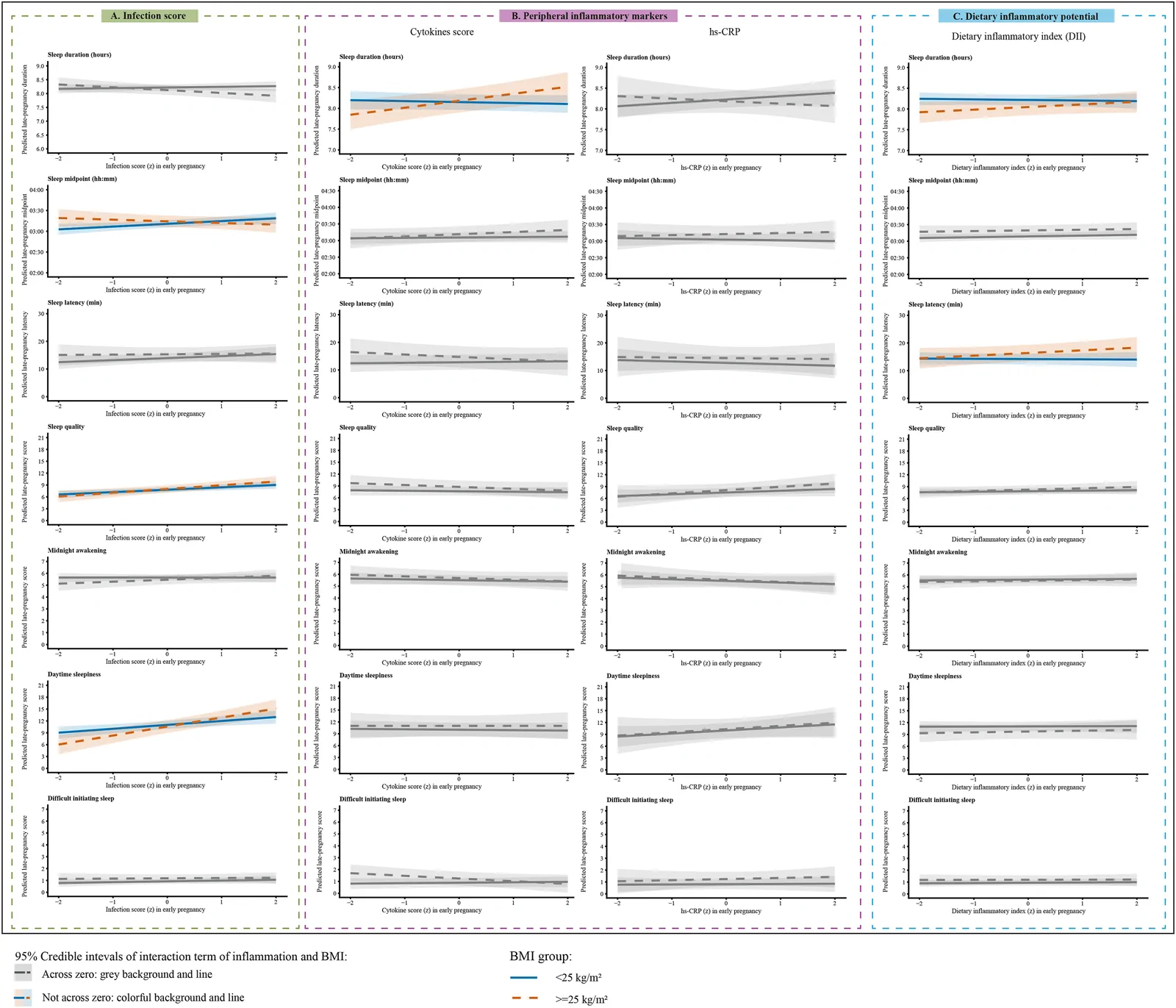
Associations between inflammation in early pregnancy and sleep in late pregnancy according to body mass index status *Figure note:* Posterior estimates (β) and 95% credible intervals (CrIs) are shown for the interaction between inflammation and body mass index (BMI) in the associations between inflammatory exposures in early pregnancy and sleep in late pregnancy. Columns represent the three inflammation-related exposures: infection score (A, green), peripheral inflammatory markers (B, purple), and dietary inflammatory potential (C, blue). Rows represent the individual sleep outcomes. If the 95% CrIs for the interaction term crossed zero, estimates are shown in grey. If the 95% CrIs did not cross zero, estimates are shown in blue and orange, representing BMI <25 kg/m^2^ and BMI ≥25 kg/m^2^, respectively. Estimates were derived from Bayesian multivariate models with seven correlated sleep components jointly specified as outcomes. Interaction terms between each inflammatory exposure in early pregnancy and BMI were included in the model, with each inflammatory exposure entered separately. The overall effect was summarized by averaging the standardized posterior regression coefficients across the seven sleep components within each posterior draw. Models were adjusted for maternal age at enrollment, maternal country of birth, maternal education, household net income per month, living with a partner, parity before enrollment, BMI, smoking before and during pregnancy, alcohol use, and illicit substance use. (For interpretation of the references to colour in this figure legend, the reader is referred to the Web version of this article.)

Several associations between self-reported infections in early pregnancy and sleep in late pregnancy differed by BMI (Fig. 4A), specifically those for sleep midpoint (interaction β = −2.84, 95%CrI = [−5.38,0.31]), sleep quality (interaction β = 0.61, 95%CrI = [0.24,0.98]), and daytime sleepiness (interaction β = 0.55, 95%CrI = [0.05,1.04]). Higher infection scores were associated with an earlier sleep midpoint among women with overweight or obesity, but with a later sleep midpoint among women without overweight or obesity. Associations with daytime sleepiness were in the same direction in both groups but were stronger among women with overweight or obesity.

### 3.2 Peripheral inflammatory markers and sleep

Neither the cytokine score (β = −0.01, 95%CrI = [−0.04,0.02]) nor hs-CRP (β = 0.02, 95%CrI = [−0.04,0.08]) in early pregnancy was associated with overall sleep in late pregnancy (Fig. 3B).

The association between cytokine score in early pregnancy and sleep duration in late pregnancy differed by BMI (interaction β = 4.60, 95% CrI = [0.84,8.35]). Higher cytokine scores were associated with longer sleep duration among women with overweight or obesity, whereas the association was weaker and in the opposite direction among women without overweight or obesity (Fig. 4B).

### 3.3 Dietary inflammatory potential and sleep

DII in early pregnancy was not associated with overall sleep in late pregnancy (β = 0.02, 95%CrI = [−0.01,0.04]) in the full sample (Fig. 3C). However, the associations of DII with sleep duration (interaction β = 3.44, 95%CrI = [0.82,5.98]) and sleep latency (interaction β = 0.65, 95%CrI = [0.03,1.27]) differed by BMI. Higher DII was associated with longer sleep duration and longer sleep latency among women with overweight or obesity, whereas associations were weaker and in the opposite direction among women without overweight or obesity (Fig. 4C).

### 3.4 Sensitivity analyses

Findings were generally similar after dichotomization of the infection score (overall sleep: β = 0.08, 95%CrI = [0.03,0.14]) and when selected sleep outcomes were refitted under alternative distributional assumptions. After additionally adjusting total energy intake in DII models, the estimates increased but the credible interval still included zero (overall sleep: β = 0.03, 95%CrI = [−0.02,0.07]).

In leave-one-component-out analyses, self-reported infections showed a similar overall association, with estimates ranging from 0.03 to 0.05 compared with 0.06 in the main analysis. Results for cytokine score were unchanged, with effect estimates remaining close to zero. For DII, leave-one-component-out analyses suggested component-dependent shifts in the overall association. Relative to the full-score estimate (β = 0.02, 95%CrI = [−0.01,0.04]), excluding thiamin increased the overall estimate (β = 0.03, 95%CrI = [−0.01,0.20]). In contrast, excluding folic acid attenuated the overall estimate towards/slightly below the null (β = −0.010, 95%CrI = [−0.31,0.05]).

### 3.5 Exploratory analyses

Self-reported infections at both preconception (n = 360, β = 0.05, 95%CrI = [0.01,0.10]) and late pregnancy (n = 1,251, β = 0.03, 95%CrI = [0.01,0.06]) were associated with poorer overall sleep in late pregnancy (Table S3). For specific sleep components, preconception self-reported infections were associated with poorer sleep quality (β = 0.64, 95%CrI = [0.11,1.16]) and a later sleep midpoint (β = 4.51, 95%CrI = [0.30,8.72]), whereas self-reported infections in late pregnancy was associated with poorer sleep quality (β = 0.41, 95%CrI = [0.12, 0.69]) and greater daytime sleepiness (β = 0.66, 95%CrI = [0.19,1.14]). These associations were generally weaker than those observed for self-reported infections in early pregnancy.

Neither the cytokine score at preconception (n = 166, β = −0.02, 95%CrI = [−0.06,0.01]), hs-CRP at preconception (n = 166, β = −0.05, 95%CrI = [−0.14,0.04]), nor the cytokine score in late pregnancy (n = 442, β = −0.001, 95%CrI = [−0.04,0.02]) was associated with overall sleep in late pregnancy (Table S3). For individual sleep components, higher preconception cytokine scores were associated with better sleep quality (β = −0.53, 95%CrI = [−0.86,-0.20]), whereas preconception hs-CRP showed a possible association with less difficulty initiating sleep, although the credible interval included zero (β = −0.43, 95%CrI = [−0.71,0.17]).

## 4 Discussion

In this population-based prospective study, inflammation-related exposures in early pregnancy showed domain-specific, component-specific, and BMI-dependent associations with sleep in late pregnancy. The clearest associations were observed for self-reported infections, particularly for sleep quality and daytime sleepiness, whereas peripheral inflammatory markers and dietary inflammatory potential were not associated with overall sleep in the full sample. Several associations were stronger among women with overweight or obesity. These findings suggest that associations between inflammation-related exposures and sleep during pregnancy may differ according to the exposure and sleep component assessed and, for some associations, according to pre-pregnancy BMI.

Among inflammation-related exposures examined, the self-reported infection score showed some of the clearest associations with poorer overall sleep in late pregnancy, particularly for sleep latency, quality, daytime sleepiness, and difficulty initiating sleep. These findings are broadly consistent with prior studies in general population (Bjorvatn et al., 2023; Prather and Leung, 2016) and prior findings in pregnant women (Amare et al., 2022; Jimah et al., 2022). Our findings extend this literature by showing associations across multiple sleep components using a composite score of infection-related conditions. The infection score may capture self-reported symptomatic conditions that can be accompanied by inflammatory responses. However, neither the occurrence of infection nor the corresponding inflammatory response was clinically or biologically verified in the present study. The observed associations should therefore be interpreted as associations with self-reported infection-related conditions rather than with clinically confirmed infection or directly measured systemic inflammation. These processes may affect sleep directly through symptoms such as discomfort and fatigue, and indirectly through “sickness behaviors” including increased daytime sleepiness and changes in sleep duration that may help conserve energy for immune defense (Ibarra-Coronado et al., 2015). Exploratory analyses further suggest that these associations may persist from preconception to late pregnancy. Although associations appeared stronger for the earlier-pregnancy infection score, differences in item composition and assessment timing across measurement periods prevent conclusions about a sensitive window.

In contrast, peripheral inflammatory marker composites in early pregnancy were not associated with later overall sleep or individual sleep components in late pregnancy, and exploratory analyses at preconception and late pregnancy likewise did not support robust associations. These null findings are consistent with one prior study showing that neither IL-6 nor CRP was related to sleep quality, but contrast with another study that reported such an association (Okun et al., 2007; Zhu et al., 2020). Our findings extend prior work by evaluating broader marker composites rather than isolated markers. Single-time-point peripheral inflammatory marker measurements capture a relatively narrow biological window and may not represent sustained inflammatory activity across the interval before the sleep assessment. This measurement difference may partly contribute to the null findings, although a true absence of association cannot be excluded.

Research on DII and sleep during pregnancy and postpartum remains sparse, with the available studies conducted among women with overweight or obesity (Tamanna et al., 2026; Wirth et al., 2022). These studies found an association between higher DII and longer sleep latency. We observed a similar association among women with overweight or obesity, but this association was not observed in the full sample. Estimates also varied across the leave-one-component-out analyses, suggesting some sensitivity to the dietary components included in the DII. These findings should therefore be interpreted specifically in relation to dietary inflammatory potential as operationalized by the DII and do not address broader dietary characteristics that may relate to sleep through metabolic, neurobiological, or circadian pathways (Eban-Rothschild et al., 2018; Haufe and Leeners, 2023).

Several associations between inflammation-related exposures and individual sleep components differed according to pre-pregnancy BMI. BMI-defined overweight and obesity are associated with differences in inflammatory regulation and with other physiological and behavioural characteristics that may modify associations between inflammation-related exposures and sleep (Mathis and Shoelson, 2011; Rigobon et al., 2018). Although the overall pattern of BMI modification was broadly consistent with amplification, associations with some sleep components differed in direction between BMI groups. This indicates that effect modification by BMI may not reflect a single underlying mechanism and may vary across sleep components. Such heterogeneity may also help contextualize the lack of clear associations in the overall sample, as averaging across BMI groups could obscure group-specific patterns. Taken together, these findings identify pre-pregnancy BMI as a potentially relevant factor to consider when examining associations between inflammation-related exposures and sleep. However, BMI alone cannot identify the underlying physiological pathways. Future studies incorporating more specific measures, such as fat distribution, insulin resistance, and repeated inflammatory biomarkers, may help determine whether adiposity-related or other pathways contribute to the observed effect modification.

A key strength is the integration of multiple inflammation-related exposures, correlated sleep components, and BMI stratification within a prospective design, allowing us to evaluate whether inflammation-related sleep vulnerability is domain-specific and context-dependent. This provides a more differentiated assessment of conceptually distinct inflammation-related exposures and sleep components within the same cohort. Several limitations should also be considered. First, four measurement-related limitations should be noted: (1) infections and sleep were assessed by self-report and may therefore be subject to reporting bias; (2) the summed scores used to represent inflammation-related exposures did not capture all relevant infectious conditions, dietary components, or cytokines, which may have attenuated associations; (3) the fever item covered the previous year and may partly reflect preconception rather than early-pregnancy infection; and (4) information on the duration, severity, clinical confirmation, and treatment of the reported conditions was unavailable. Second, Inflammation and sleep may be bidirectionally related (Engert and Besedovsky, 2025). However, comparable repeated assessments of both processes were unavailable across gestation, preventing us from examining the reverse direction or bidirectional associations. Third, residual confounding cannot be excluded, including factors not measured in this study, such as physical activity. Fourth, the relatively high socioeconomic status of the sample may limit generalizability. Finally, the observational study design did not establish causality.

In conclusion, inflammation-related exposures in early pregnancy showed heterogeneous associations with sleep in late pregnancy, with the clearest evidence observed for self-reported infections and for sleep quality and daytime sleepiness. These findings support examining distinct inflammation-related exposures and sleep components separately and suggest that some associations may differ according to pre-pregnancy BMI.

## CRediT authorship contribution statement

**Fangxiang Mao:** Conceptualization, Data curation, Formal analysis, Funding acquisition, Methodology, Software, Validation, Visualization, Writing – original draft, Writing – review & editing. **Milan Zarchev:** Funding acquisition, Writing – review & editing. **Hong Sun:** Formal analysis, Funding acquisition, Methodology, Writing – review & editing. **Yuchan Mou:** Methodology, Writing – review & editing. **Romy Gaillard:** Methodology, Writing – review & editing. **Isabel K. Schuurmans:** Conceptualization, Funding acquisition, Methodology, Supervision, Writing – review & editing. **Hanan El Marroun:** Conceptualization, Funding acquisition, Methodology, Supervision, Writing – review & editing.

## Ethics declaration

Written informed consent to take part in the study and to publish the article has been obtained from all participants or their legal representatives. The privacy rights of participants have been observed.

This study was performed in compliance with relevant laws, regulatory frameworks and guidelines where the research took place. This study was approved by the Medical Ethical Committee of the Erasmus Medical Center. (Approval No. MEC-2016-589)

## Data availability statement

The analysis plan, statistical code, and data from this study are available upon request to the Management Team of the Generation R Study (generationr@erasmusmc.nl), subject to local, national, and European rules and regulations.

## Funding sources

This work was supported by the Erasmus Medical Center and the Erasmus University Rotterdam, the Netherlands Organization for Health Research and Development (ZonMW), the Netherlands Organisation for Scientific Research (NWO), the Ministry of Health, Welfare and Sport and the Ministry of Youth and Families. This work is part of the *HappyMums* project funded by the European Union (Grant Agreement No. 101057390) with HM and IS supported by this grant. The work of IS is further supported by the European Research Council (TEMPO; Grant Agreement No. 101039672). FM received a scholarship from China Scholarship Council (CSC, 202206220040). HM was supported by Stichting Volksbond Rotterdam, the Netherlands Organization for Health Research and Development [Aspasia grant No.015.016.056]. MZ received funding from NIH grant (No. R01MH124776). HS received a scholarship from China Scholarship Council (CSC, 202206240031). RG received funding from the Dutch Diabetes Foundation (Grant No. 2024.28.001), from the Netherlands Organization for Health Research and Development (NWO, ZonMW, grant number 05430052110007, and NWO, ZonMw VIDI Grant 09150172110034), and from the European Union (ERC, OBESE-EMBRYO, ERC-2024-STG-101161004).

These funding sources are not involved in the study design, collection, analysis and interpretation of data, writing of the report and decision to submit the article for publication.

## Declaration of competing interest

The authors declare that they have no known competing financial interests or personal relationships that could have appeared to influence the work reported in this paper.
