# Supplementary material for: Inflammatory exposures in early pregnancy and multidimensional sleep in late pregnancy: a prospective population-based study
Source: Brain Behav Immun Health. 2026 Sep 9;57:101354. doi: 10.1016/j.bbih.2026.101354 (PMC13583606; doi:10.1016/j.bbih.2026.101354)
Supplement: Supplementary file 1 [file mmc1.docx]

**Supplementary materials**

Supplementary methods: Calculation of dietary inflammatory index

Supplementary tables

**Supplementary methods: Calculation of dietary inflammatory index**

We quantified dietary inflammatory potential using the Dietary Inflammatory Index (DII), a literature-derived, population-based score developed by Shivappa et al. (1, 2). The DII incorporates 45 food items, for which global mean daily intake and standard deviations were obtained from 11 international datasets. Each parameter is assigned an inflammatory effect score according to the number and quality of studies linking it to peripheral inflammatory markers.

The maternal DII in our study was assessed in early pregnancy and calculated based on food items derived from the semi-quantitative HELIUS (Healthy Life in an Urban Setting) food frequency questionnaire (FFQ) (3). The Dutch version of the HELIUS FFQ encompasses 238 food items. Mothers were asked to report the frequency of consumption of these food items over the past 4 weeks. We excluded participants with implausible habitual dietary intake, defined as energy intake <500 or >3500 kcal per day for women (2.7%).

In our study, 26 of the original 45 DII food parameters were available: alcohol, vitamin B12, vitamin B6, β-carotene, caffeine, carbohydrate, energy, total fat, fiber, folic acid, iron, magnesium, monounsaturated fatty acids, n-3 fatty acid, n-6 fatty acid, onion (including onion and green onion), protein, polyunsaturated fatty acids, riboflavin, saturated fat, thiamin, trans fat, vitamin A, vitamin C, vitamin D, zinc, and tea (Table S1). The DII was calculated in four steps:

1. Standardize intake of each food parameter by calculating z scores using global means and standard deviations.
2. Convert z scores to percentiles using the cumulative distribution function, then create zero-centered percentiles by doubling the percentile and subtracting 1 (zero-centered percentile = percentile × 2 − 1).
3. Multiply each zero-centered percentile by its food parameter’s inflammatory effect score.
4. Sum the 26 parameter-specific scores to obtain an overall DII score for each participant.

A higher DII score indicates a more pro-inflammatory diet, whereas a lower score indicates a more anti-inflammatory diet. We used the R package ‘Dietaryindex’ to calculate the score (4).

Table S1: Food parameters used to calculate the Dietary Inflammatory Index

| **Food parameters** | **Global daily mean intake** | **Global standard deviation** | **Inflammatory effect score** |
| --- | --- | --- | --- |
| Alcohol (g) | 13.98 | 3.72 | -0.278 |
| Vitamin B12 (μg) | 5.15 | 2.70 | 0.106 |
| Vitamin B6 (mg) | 1.47 | 0.74 | -0.365 |
| β-Carotene (μg) | 3718.00 | 1720.00 | -0.584 |
| Caffeine (g) | 8.05 | 6.67 | -0.110 |
| Carbohydrate (g) | 272.20 | 40.00 | 0.097 |
| Energy (kcal) | 2056.00 | 338.00 | 0.180 |
| Total fat (g) | 71.40 | 19.40 | 0.298 |
| Fiber (g) | 18.80 | 4.90 | -0.663 |
| Folic acid (μg) | 273.00 | 70.70 | -0.190 |
| Fe (mg) | 13.35 | 3.71 | 0.032 |
| Mg (mg) | 310.10 | 139.40 | -0.484 |
| MUFA (g) | 27.00 | 6.10 | -0.009 |
| n-3 Fatty acids (g) | 1.06 | 1.06 | -0.436 |
| n-6 Fatty acids (g) | 10.800 | 7.50 | -0.159 |
| Onion (g) | 35.90 | 18.40 | -0.301 |
| Protein (g) | 79.40 | 13.90 | 0.021 |
| PUFA (g) | 13.88 | 3.76 | -0.337 |
| Riboflavin (mg) | 1.70 | 0.79 | -0.068 |
| Saturated fat (g) | 28.60 | 8.00 | 0.373 |
| Thiamin (mg) | 1.70 | 0.66 | -0.098 |
| Trans fat (g) | 3.15 | 3.75 | 0.229 |
| Vitamin A (RE) | 983.90 | 518.6 | -0.401 |
| Vitamin C (mg) | 118.20 | 43.46 | -0.424 |
| Zn (mg) | 9.84 | 2.19 | -0.313 |
| Green/black tea (g) | 1.69 | 1.53 | -0.536 |

*Table note:* Global daily mean intake, global standard deviation, and the inflammatory effect score were derived from the original Dietary Inflammatory Index developed by Shivappa et al. (2).

Abbreviations: g, gram; mg, milligram; µg, microgram; kcal, kilocalorie; RE, retinol equivalents; MUFA, monounsaturated fatty acids; PUFA, polyunsaturated fatty acids.

**References**

1. Shivappa N, Steck SE, Hurley TG, Hussey JR, Ma Y, Ockene IS, *et al.* (2014): A population-based dietary inflammatory index predicts levels of C-reactive protein in the Seasonal Variation of Blood Cholesterol Study (SEASONS). *Public Health Nutr* 17: 1825–1833.

2. Shivappa N, Steck SE, Hurley TG, Hussey JR, Hébert JR (2014): Designing and developing a literature-derived, population-based dietary inflammatory index. *Public Health Nutr* 17: 1689–1696.

3. Beukers MH, Dekker LH, de Boer EJ, Perenboom CWM, Meijboom S, Nicolaou M, *et al.* (2015): Development of the HELIUS food frequency questionnaires: ethnic-specific questionnaires to assess the diet of a multiethnic population in The Netherlands. *Eur J Clin Nutr* 69: 579–584.

4. Zhan JJ, Hodge RA, Dunlop AL, Lee MM, Bui L, Liang D, Ferranti EP (2024): Dietaryindex: a user-friendly and versatile R package for standardizing dietary pattern analysis in epidemiological and clinical studies. Am J Clin Nutr 120: 1165–1174

**Supplementary tables**

Table S2: Characteristics of nonresponders and responders

|  | **Nonresponder (n=841)** | **Responder**  **(n=1,216)** | **p-value** |
| --- | --- | --- | --- |
| **Sociodemographic characteristics** |  |  |  |
| Age at study enrollment, mean ± SD | 31.7 ± 4.0 | 32.0 ± 4.1 | 0.300 |
| Country of birth |  |  | 0.700 |
| The Netherlands | 541 (67.0%) | 796 (66.2%) |  |
| Outside the Netherlands | 266 (33.0%) | 406 (33.8%) |  |
| Education, No. (%) |  |  | <0.001 |
| Prevocational or vocational | 228 (28.7%) | 266 (22.3%) |  |
| Bachelor’s or associate degree | 256 (32.2%) | 355 (29.7%) |  |
| Master’s degree | 311 (39.1%) | 574 (48.0%) |  |
| Household net income, monthly, No. (%) |  |  | 0.006 |
| Less than €3000 | 176 (24.0%) | 197 (18.6%) |  |
| More than €3000 | 557 (76.0%) | 863 (81.4%) |  |
| Living with partner, No. (%) |  |  | 0.300 |
| Yes | 674 (86.9%) | 1,047 (88.5%) |  |
| No or Other | 102 (13.1%) | 136 (11.5%) |  |
| Parity before enrollment, No. (%) |  |  | <0.001 |
| 0, nulliparous | 463 (58.5%) | 838 (70.1%) |  |
| ≥1, multiparous | 329 (41.5%) | 357 (29.9%) |  |
|  |  |  |  |
| **Psychosocial and lifestyle characteristics** |  |  |  |
| Body mass index at preconception (kg/m^2^), mean ± SD | 24.1 ± 4.4 | 24.0 ± 4.3 | 0.700 |
| Smoking before and during pregnancy, No. (%) |  |  | 0.056 |
| Never | 433 (57.0%) | 615 (56.7%) |  |
| Yes, quit >3 months before pregnancy | 163 (21.4%) | 243 (22.4%) |  |
| Yes, quit <3 months before pregnancy | 43 (5.7%) | 81 (7.5%) |  |
| Yes, quit smoking in the first trimester | 77 (10.1%) | 110 (10.1%) |  |
| Yes, smoked during the whole pregnancy | 44 (5.8%) | 35 (3.2%) |  |
| Alcohol use in the past 12 months, No. (%) |  |  | 0.700 |
| None | 159 (20.8%) | 235 (20.1%) |  |
| Any | 604 (79.2%) | 933 (79.9%) |  |
| Illicit substance use in the past 12 months, No. (%) |  |  | 0.700 |
| None | 765 (91.1%) | 1,100 (90.5%) |  |
| Any | 75 (8.9%) | 116 (9.5%) |  |

*Table Note:* Among 2,057 eligible pregnancies invited to the late-pregnancy assessment, 1,216 (59.1%) contributed data on at least one sleep or inflammation measure. Baseline characteristics are compared between responders and nonresponders. Values are presented as mean ± standard deviation (SD) or n (%), as appropriate.

Table S3: Inflammation at preconception and in late pregnancy in relation to sleep in late pregnancy

| **Sleep components** | **Infection score** | |  | **Peripheral inflammatory markers** | | | |
| --- | --- | --- | --- | --- | --- | --- | --- |
|  | **At preconception (n=360), β [95% CrIs]** | **In late pregnancy (n=1,261), β [95% CrIs]** |  | **At preconception cytokine score (n=166), β [95% CrIs]** | **In late pregnancy cytokine score (n=442), β [95% CrIs]** |  | **At preconception**  **hs-CRP (n=166), β [95% CrIs]** |
| Sleep duration | -1.61 [-6.55, 3.29] | 0.11 [-3.03, 3.29] |  | 0.40 [-2.90, 3.71] | 0.91 [-2.05, 3.90] |  | 1.27 [-7.54, 10.11] |
| Sleep midpoint | **4.51 [0.30, 8.72]** | 1.31 [-1.17, 3.80] |  | 0.27 [-2.67, 3.23] | 1.32 [-1.19, 3.85] |  | 0.02 [-7.97, 8.10] |
| Sleep latency | 0.42 [-0.88, 1.75] | 0.10 [-0.65, 0.86] |  | -0.10 [-0.93, 0.72] | -0.02 [-0.73, 0.7] |  | -1.58 [-3.75, 0.61] |
| Sleep quality | **0.64 [0.11, 1.16]** | **0.41 [0.12, 0.69]** |  | **-0.53 [-0.86, -0.20]** | -0.19 [-0.49, 0.10] |  | -0.16 [-1.08, 0.77] |
| Midnight awakenings | 0.01 [-0.21, 0.23] | 0.09 [-0.02, 0.20] |  | -0.01 [-0.15, 0.13] | -0.08 [-0.19, 0.03] |  | -0.23 [-1.72, 1.25] |
| Daytime sleepiness | 0.82 [-0.02, 1.67] | **0.66 [0.19, 1.14]** |  | -0.31 [-0.86, 0.24] | -0.08 [-0.55, 0.40] |  | 0.11 [-0.30, 0.53] |
| Difficulty initiating sleep | 0.06 [-0.12, 0.23] | 0.02 [-0.08, 0.11] |  | -0.05 [-0.18, 0.07] | -0.02 [-0.12, 0.09] |  | **-0.43 [-0.71, -0.17]** |
| *Overall sleep* | **0.05 [0, 0.10]** | **0.03 [0.01, 0.06]** |  | -0.02 [-0.06, 0.01] | -0.001 [-0.04, 0.02] |  | -0.05[-0.14, 0.04] |

*Table Note:* Posterior estimates (β) and 95% credible intervals (CrIs) are shown for associations of self-reported infections and peripheral inflammatory markers assessed at preconception or in late pregnancy with sleep in late pregnancy. Dietary inflammatory potential was not available at preconception, and hs-CRP was not available in late pregnancy. Estimates were obtained from Bayesian multivariate models with seven correlated sleep components jointly specified as outcomes, with each inflammatory exposure entered separately. The overall effect was summarized by averaging standardized posterior regression coefficients across the seven sleep components within each posterior draw.

Models were adjusted for maternal age at enrollment, maternal country of birth, maternal education, household net income per month, living with a partner, parity before enrollment, body mass index, smoking before and during pregnancy, alcohol use, and illicit substance use.
